# Supplementary material for: Locally organised and activated Fth1hi neutrophils aggravate inflammation of acute lung injury in an IL-10-dependent manner
Source: Nat Commun. 2022 Dec 13;13:7703. doi: 10.1038/s41467-022-35492-y (PMC9745290; doi:10.1038/s41467-022-35492-y)
Supplement: Supplementary file 1 — Supplementary Information [file 41467_2022_35492_MOESM1_ESM.pdf]

Supplementary Materials for

Locally Orchestrated Fth1^{hi} Neutrophils Aggravate Inflammation of Acute Lung Injury in an IL-10-Dependent Manner

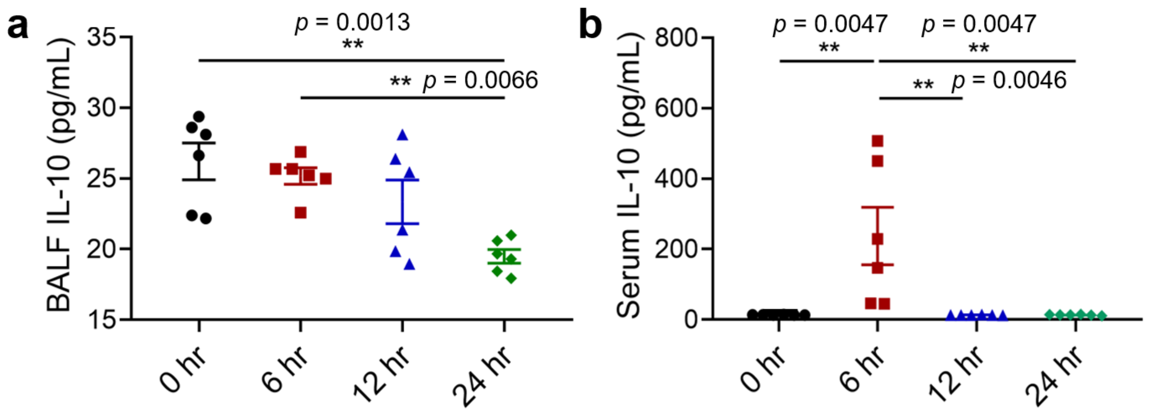

Supplementary Figure 1. **a, b** IL-10 level in BALF supernatant (**a**) and serum (**b**) of ALI mice at different time points upon LPS treatment. n = 6 per group. All samples were biologically independent and three or more independent experiments with similar results were performed. Data are presented as mean ± SEM and analyzed with a 95% confidence interval. Statistical analysis was performed using one-way ANOVA followed by Bonferroni's post hoc test. Source data are provided as a Source Data file.

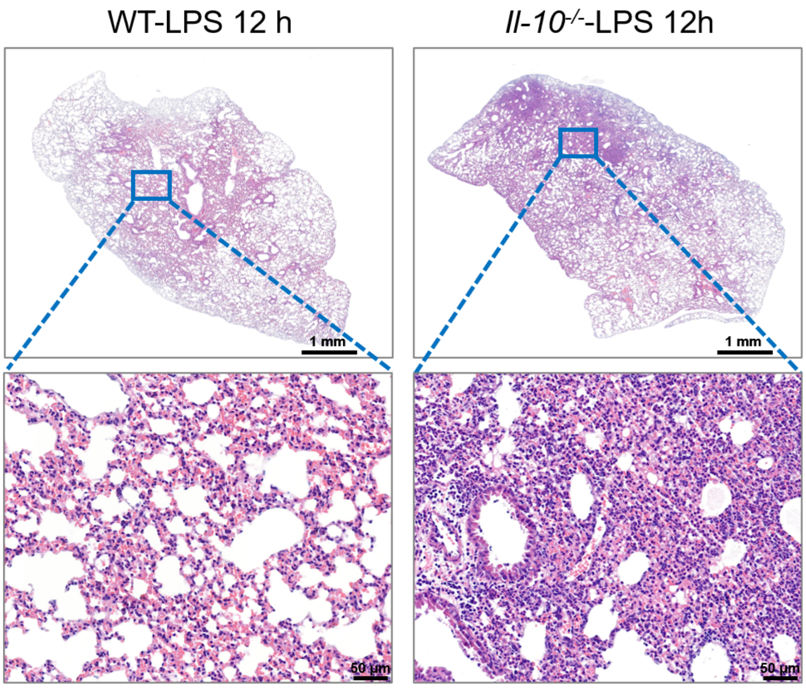

Supplementary Figure 2. Representative histological images of H&E-stained lung sections from WT and *Il-10*$^{-/-}$ mice at 12 h after LPS inhalation. Scale bars in the upper panel, 1 mm; scale bars in the below panel, 50 µm. Three independent experiments with similar results were performed.

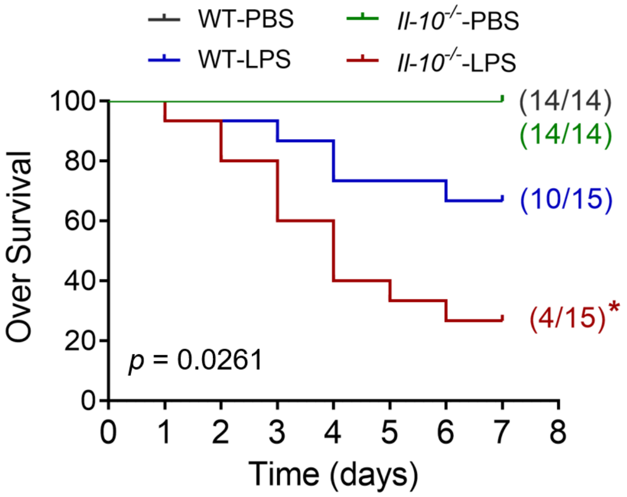

Supplementary Figure 3. The effects of *Il-10*$^{-/-}$ comparing to WT on survival of the severe ALI mice. n = 14, 14, 15, 15, respectively in each group. *p = 0.0261 versus WT-LPS group. All samples were biologically independent and three or more independent experiments with similar results were performed. Data are analyzed with a 95% confidence interval. Statistical analysis was performed using Log-rank test. Source data are provided as a Source Data file.

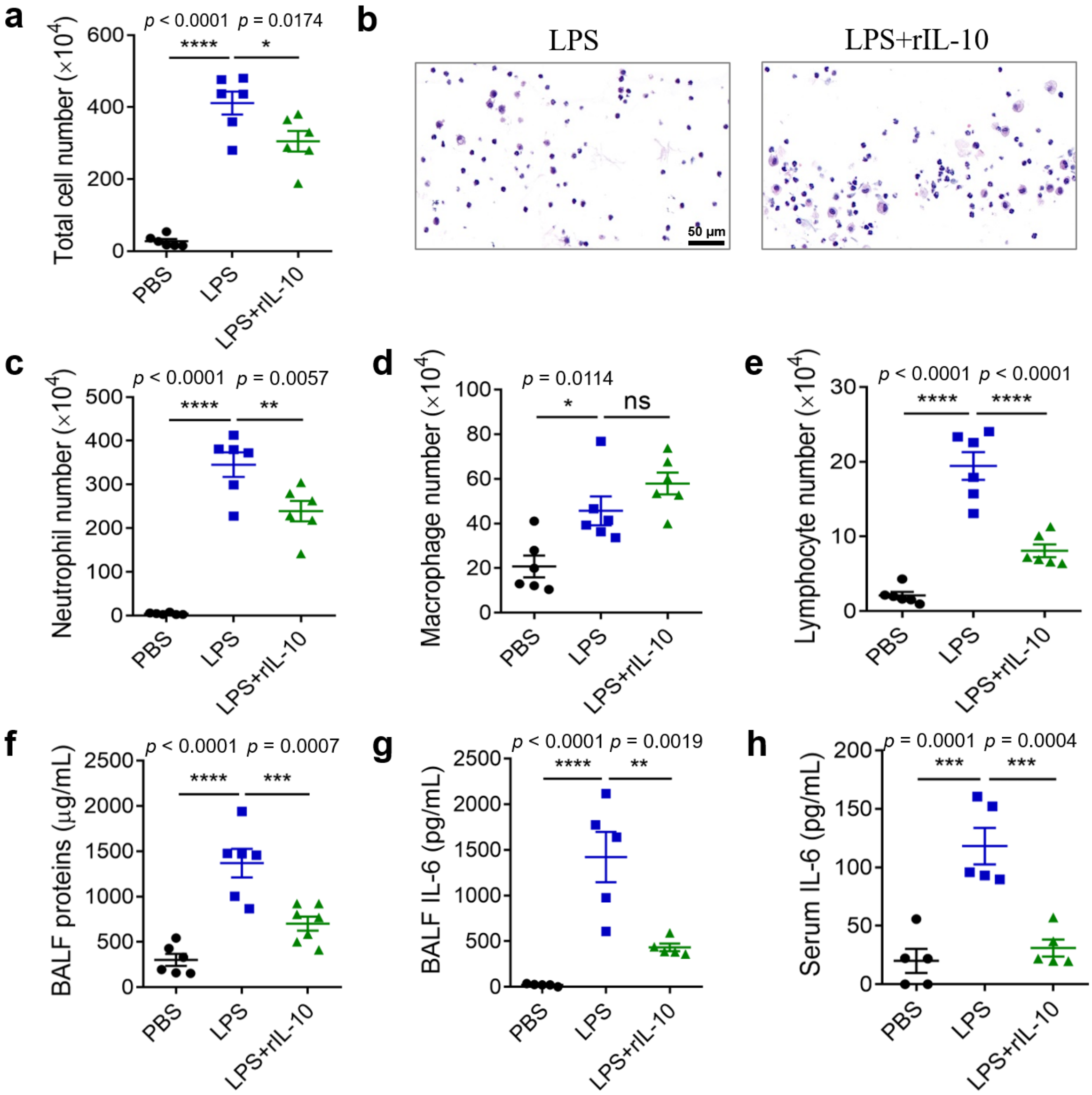

Supplementary Figure 4. a-f Mice were co-injected nasally with PBS or recombinant IL-10 (rIL-10; 45 μg/kg) together with LPS (10 mg/kg). Animals were euthanized 24 h after LPS stimulation for the analysis of total cell counts (**a**, n = 6 per group), H&E staining of cells (**b**, scale bars, 50 μm), neutrophil numbers (**c**, n = 6 per group), macrophage counts (**d**, n = 6 per group), lymphocyte numbers (**e**, n = 6 per group) and total protein in BALF (**f**, n = 6, 6, 7 in each group). **g-h** Production of IL-6 in BALF (**g**) and serum (**h**) is shown, n = 5 per group. ns, not significant. All samples were biologically independent and three or more independent experiments with similar results were performed. Data are presented as mean ± SEM and analyzed with a 95% confidence interval. Statistical analysis was performed using one-way ANOVA followed by Bonferroni's post hoc test. Source data are provided as a Source Data file.

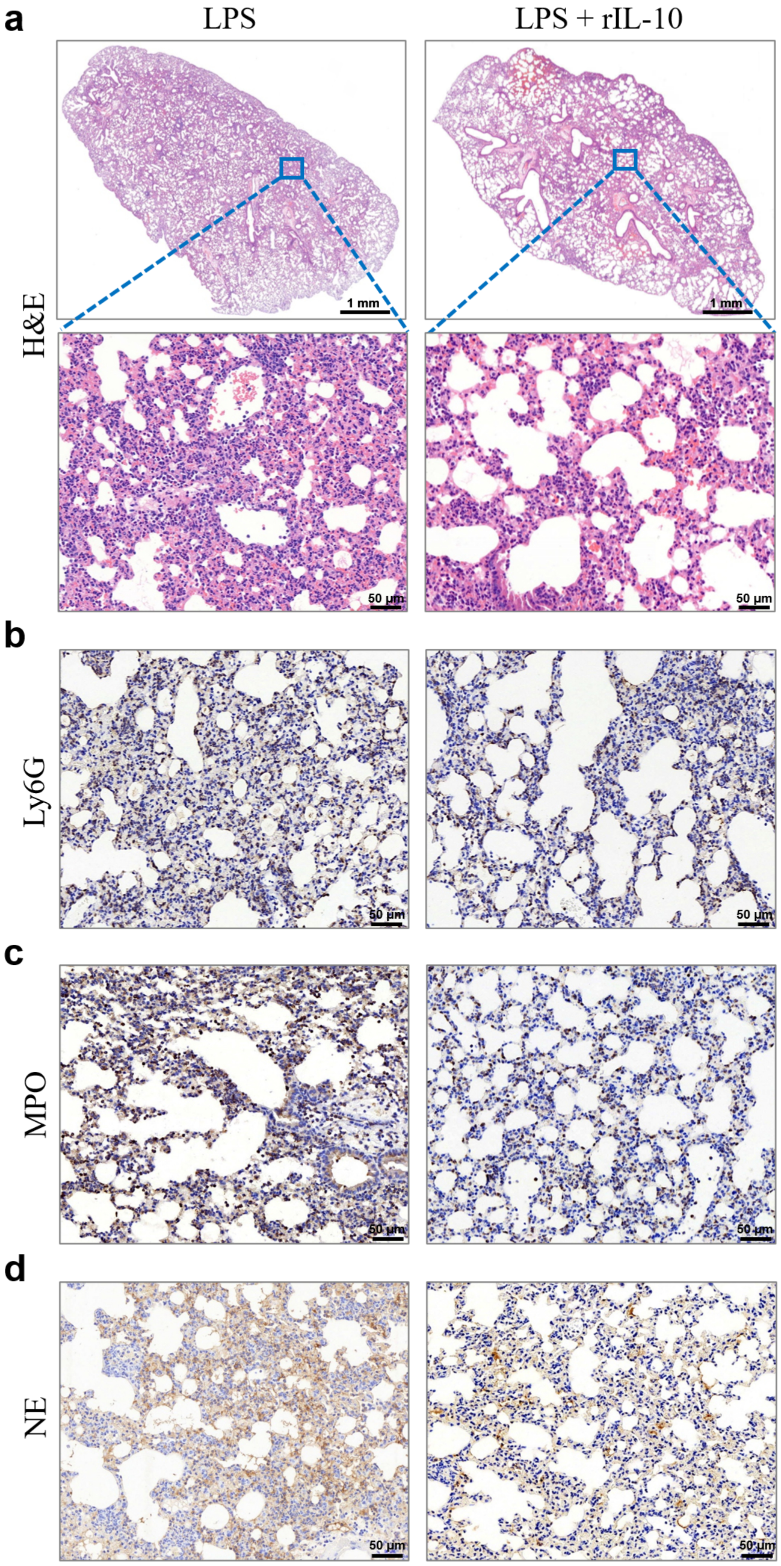

Supplementary Figure 5. **a** Representative histological images of H&E-stained lung sections at 24 h after LPS inhalation in the presence or absence of rIL-10 administration. Scale bars in the upper panel, 1 mm; scale bars in the below panel, 50 μm. **b-d** Immunohistochemical staining with Ly6G (**b**), MPO (**c**) and NE (**d**) in mouse lung sections. Scale bars, 50 μm. Three independent experiments with similar results were performed.

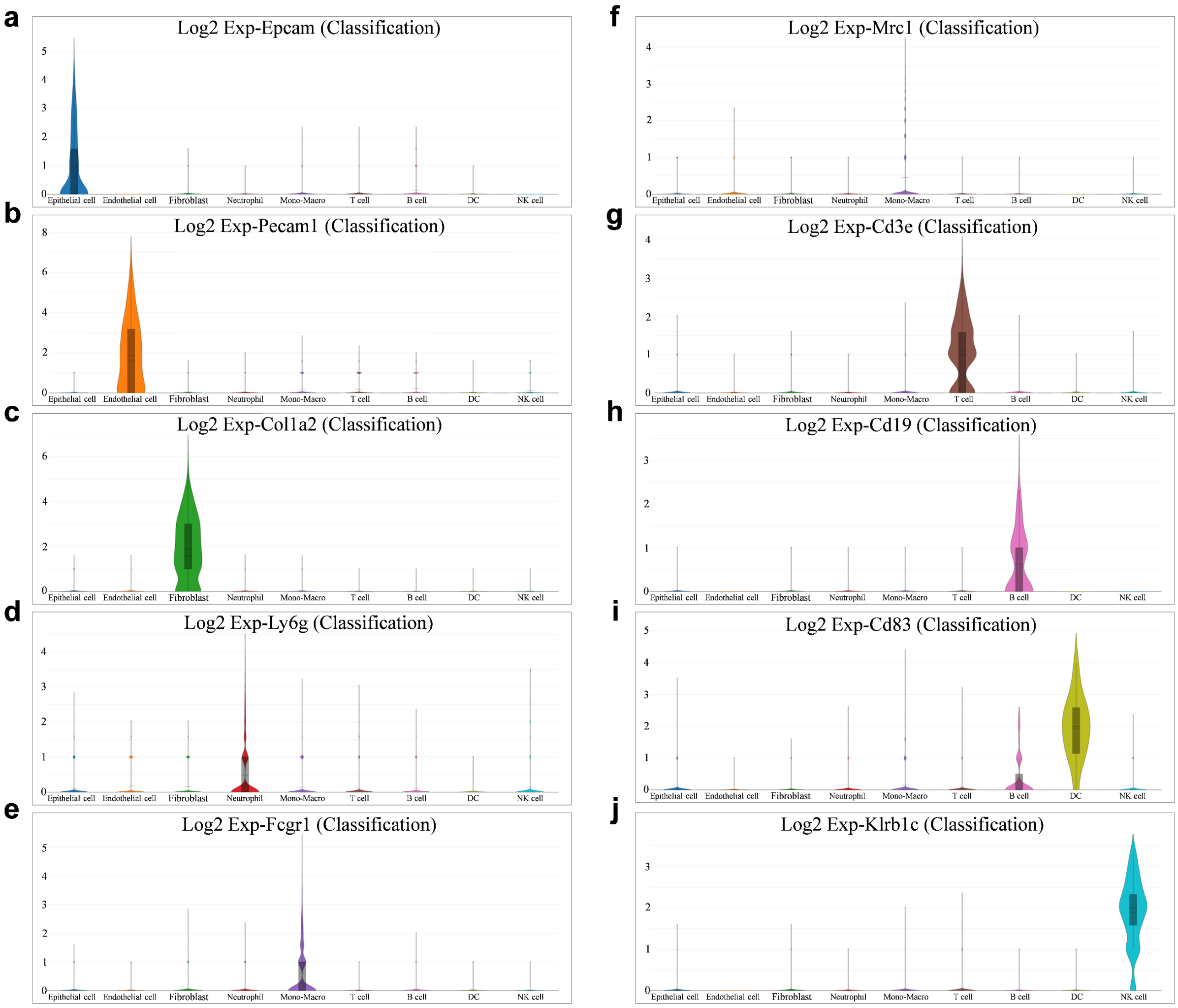

Supplementary Figure 6. **a-j** Violin plots of gene expression in single cells of each population from lungs of WT control, WT LPS-treated and *Il-10*$^{-/-}$ LPS-treated mice for described markers in the literature for epithelial cells (**a**), endothelial cells (**b**), fibroblasts (**c**), neutrophils (**d**), monocyte-macrophages (Mono-Macro) (**e, f**), lymphocyte T-cells (**g**), lymphocyte B-cells (**h**), dendritic cells (DC) (**i**) and natural killer (NK) cells (**j**). Boxes within violin plot show the median ± 1 quartile, with the whiskers extending from the hinge to the smallest or largest value within 1.5 × interquartile range from the box boundaries. *n* = 469, 168, 683, 7071, 2618, 566, 140, 55, 100, respectively.

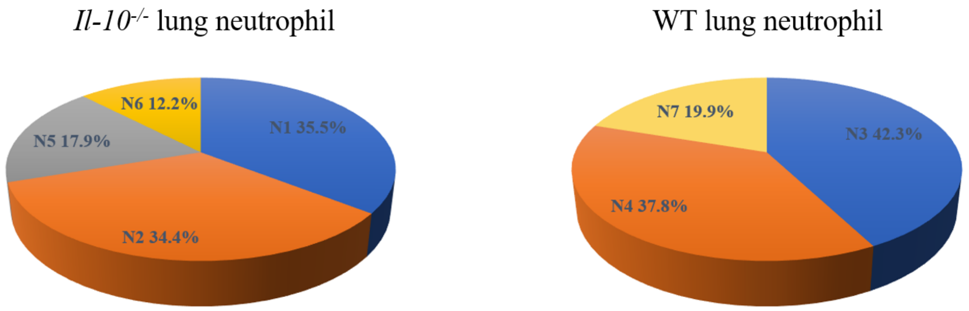

Supplementary Figure 7. Proportions of neutrophil populations in lung tissues of *Il-10*$^{-/-}$ (left) and WT (right) mouse at 24 h after LPS stimulation.

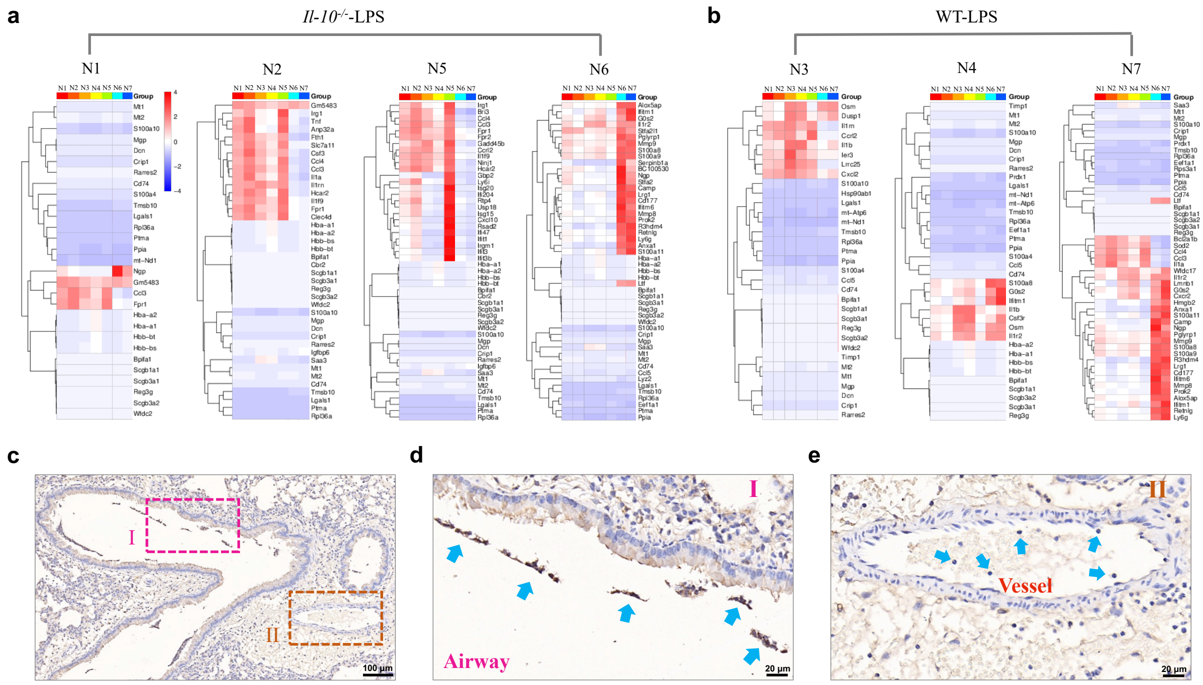

Supplementary Figure 8. **a, b** Heatmaps of the top differentially expressed genes in each neutrophil population compared to the others. **c-e** Immunohistochemical staining was performed for Fth1 to determine the distribution of the Fth1^{hi} Neu subset in ALI lung tissues. Scale bars, 100 µm (**c**); scale bars, 20 µm (**d, e**). Blue arrowheads indicate neutrophils in airways (**d**) and pulmonary vessels (**e**). Three independent experiments with similar results were performed.

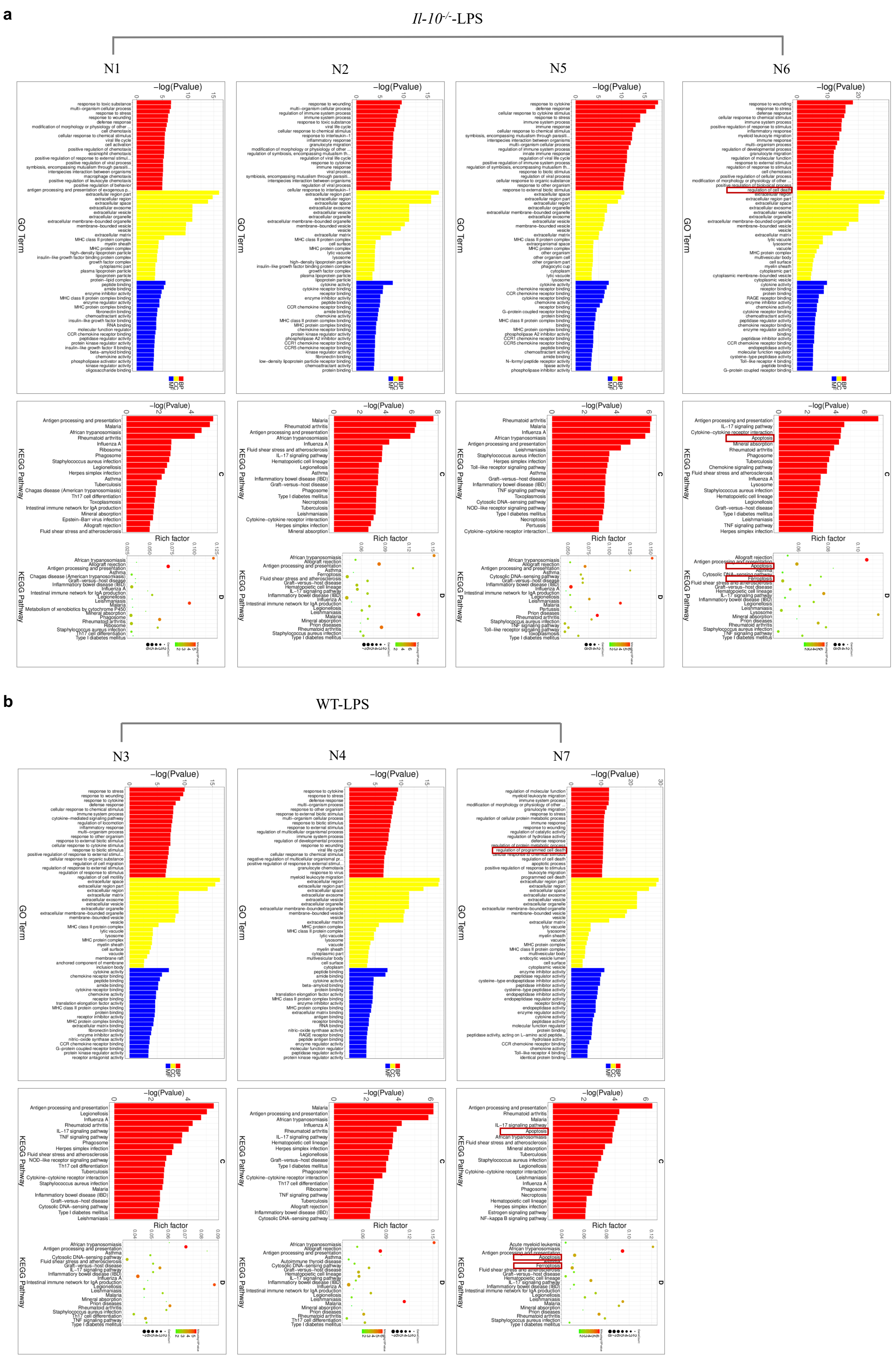

Supplementary Figure 9. **a, b** GO enrichment and KEGG pathway analyses of individual neutrophil subsets in ALI lungs from *Il-10*$^{-/-}$ (**a**) and WT (**b**) mice. Red boxes indicate the

differential functions between Fth1^{hi} (N1-N5) and Prok2^{hi} (N6 and N7) neutrophils. The statistical analysis was performed by Fisher's test.

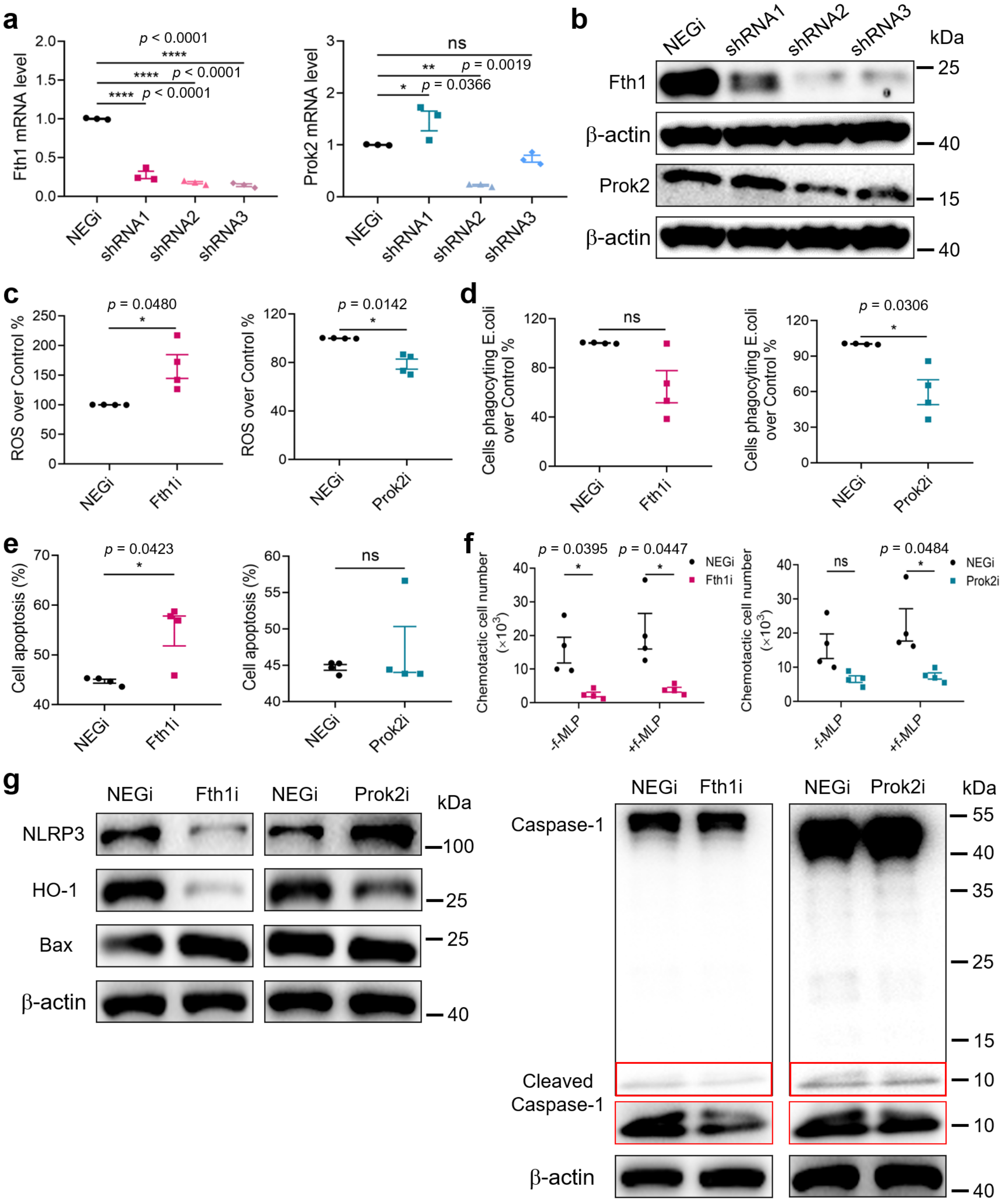

Supplementary Figure 10. **a, b** Transfection of human promyelocytic leukemia (HL-60) cells with Fth1/Prok2 shRNA downregulated the mRNA (**a**) and protein (**b**) expression at 12 h compared with control shRNA treatment. *n* = 3 per group. **c-f** Fth1-depleted neutrophils exhibited function defects in anti-oxidation (**c**), anti-apoptosis (**e**) and chemotaxis (**f**), while Prok2-deficient neutrophils defected in ROS production (**c**), phagocytosis (**d**) and chemotaxis (**f**) of HL-60 cell-derived neutrophils compared with NEGi. *n* = 4 per group. (**g**) Immunoblotting verifying the effects of Fth1/Prok2 on anti-oxidant HO-1 and pro-apoptotic Bax expression, as well as NLRP3 and cleaved Caspase-1 level for inflammasome activation.

ns, not significant. All samples were biologically independent and three or more independent experiments with similar results were performed. Data are presented as mean ± SEM and analyzed with a 95% confidence interval. Statistical analysis was performed using two-tailed unpaired Student t test. Source data are provided as a Source Data file. Uncropped scans of western blot with molecular weight markers were provided in Supplementary Figure 16.

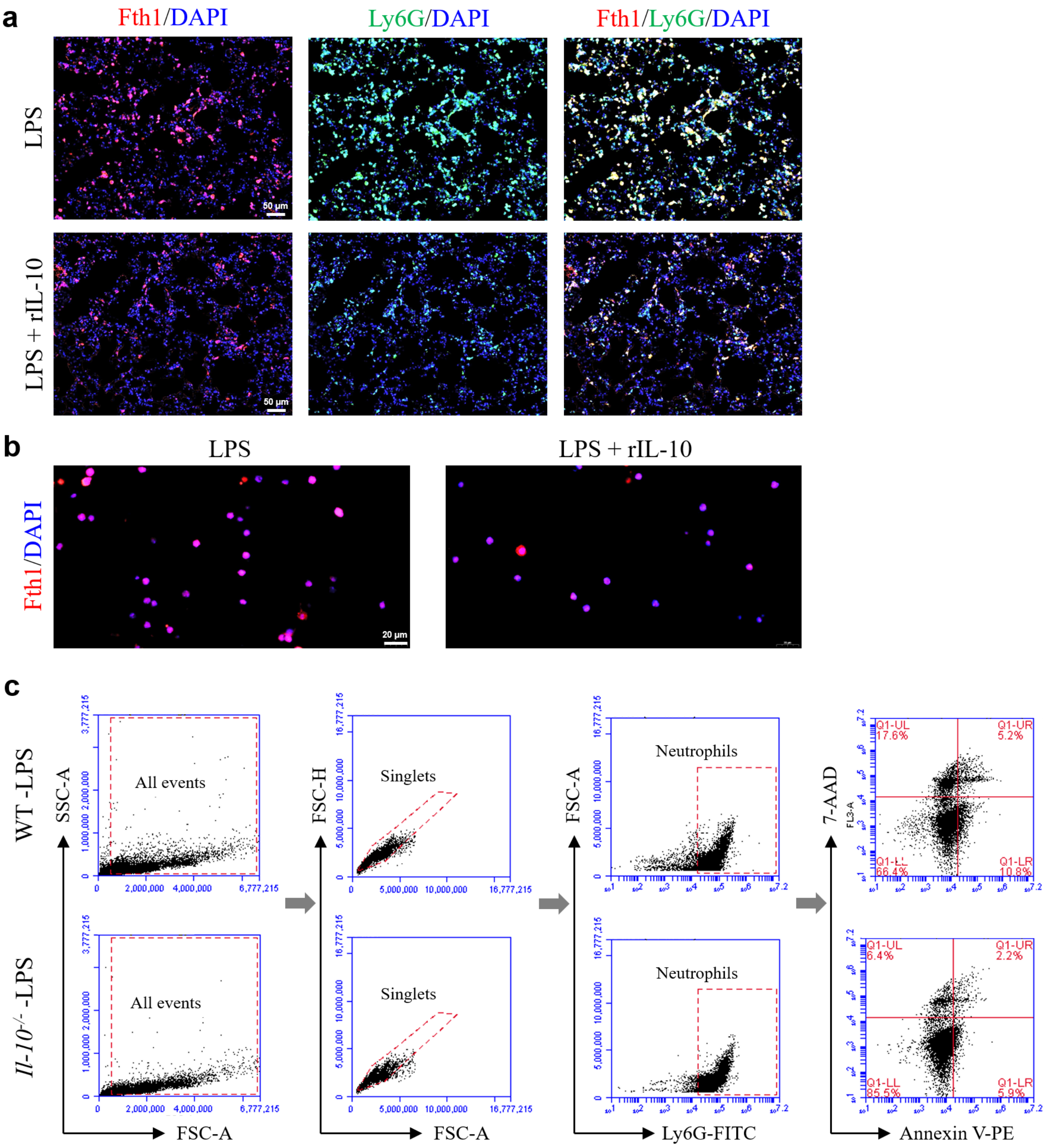

Supplementary Figure 11. **a** Representative images of anti-Fth1/anti-Ly6G immunofluorescence-stained lung sections of mice with or without rIL-10 treatment 24 h after LPS challenge. The nuclei were stained with DAPI and are displayed in blue. Scale bars, 50 µm. **b** Representative images of anti-Fth1 immunofluorescence-stained airway neutrophils of mice with or without rIL-10 administration 24 h after LPS stimulation. The nuclei were stained with DAPI, displayed in blue. Scale bars, 20 µm. Three independent experiments with similar results were performed. **c** Gating strategy and representative scatter plots of BALF cells collected from WT and *Il-10*$^{-/-}$ mice at 24 h after LPS challenge. Cellular apoptosis was evaluated by flow cytometry.

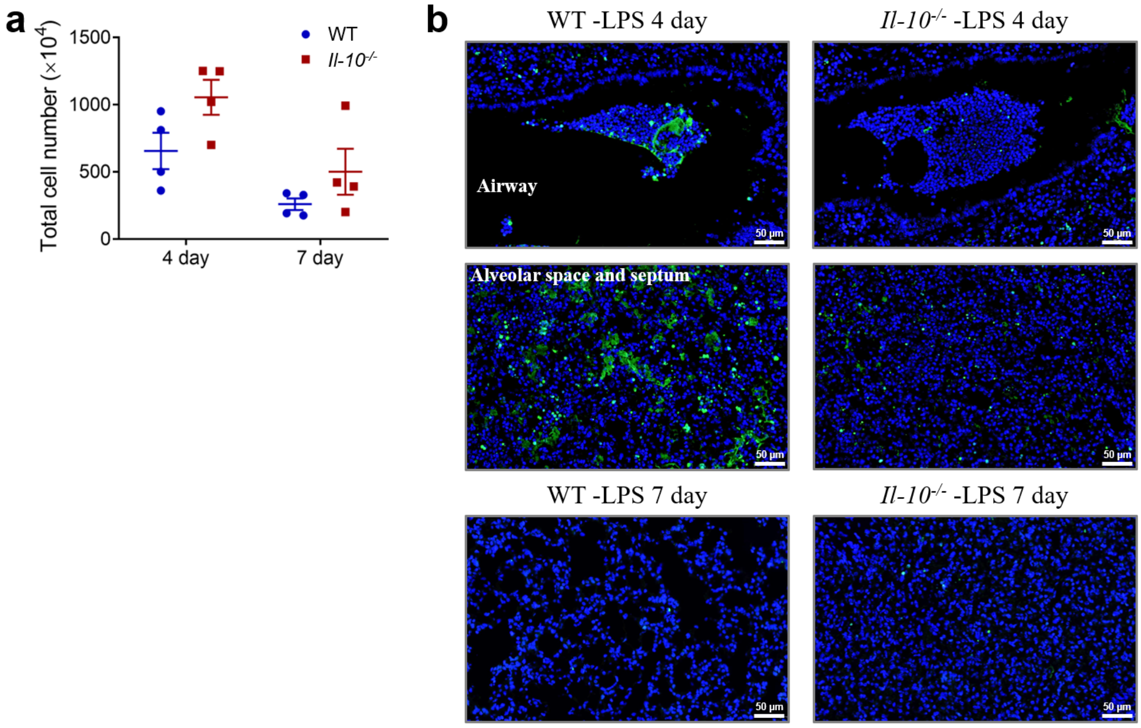

Supplementary Figure 12. **a** BALF was collected from WT and *Il-10*$^{-/-}$ mice for identifying total counts of inflammatory cells on day 4 and day 7 after LPS challenge, $n = 4$ in each group. **b** TUNEL staining of apoptotic cells (green) and DAPI staining of nuclei (blue) in lung sections at 4 d and 7 d post-exposure. ns, not significant. All samples were biologically independent and three or more independent experiments with similar results were performed. Data are presented as mean ± SEM and analyzed with a 95% confidence interval. Statistical analysis was performed using two-tailed unpaired Student t test. Source data are provided as a Source Data file.

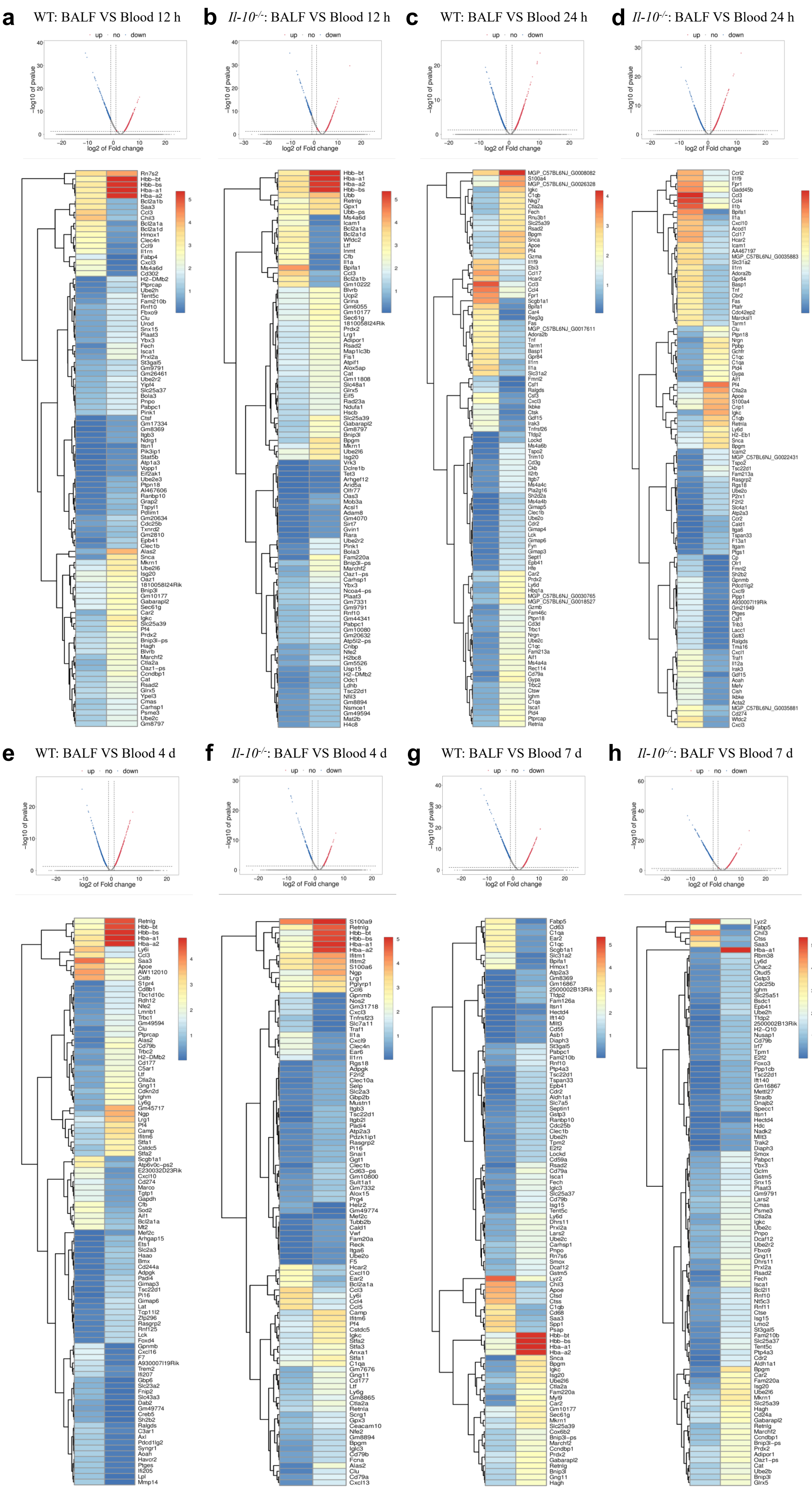

Supplementary Figure 13. **a-h** The volcano plots (upper) and heatmaps (below) showing the

differentially expressed genes in BALF and blood neutrophils from distinct management groups at different time points. n = 4 samples per group. VS, versus. p values were calculated with a likelihood ratio test between groups.

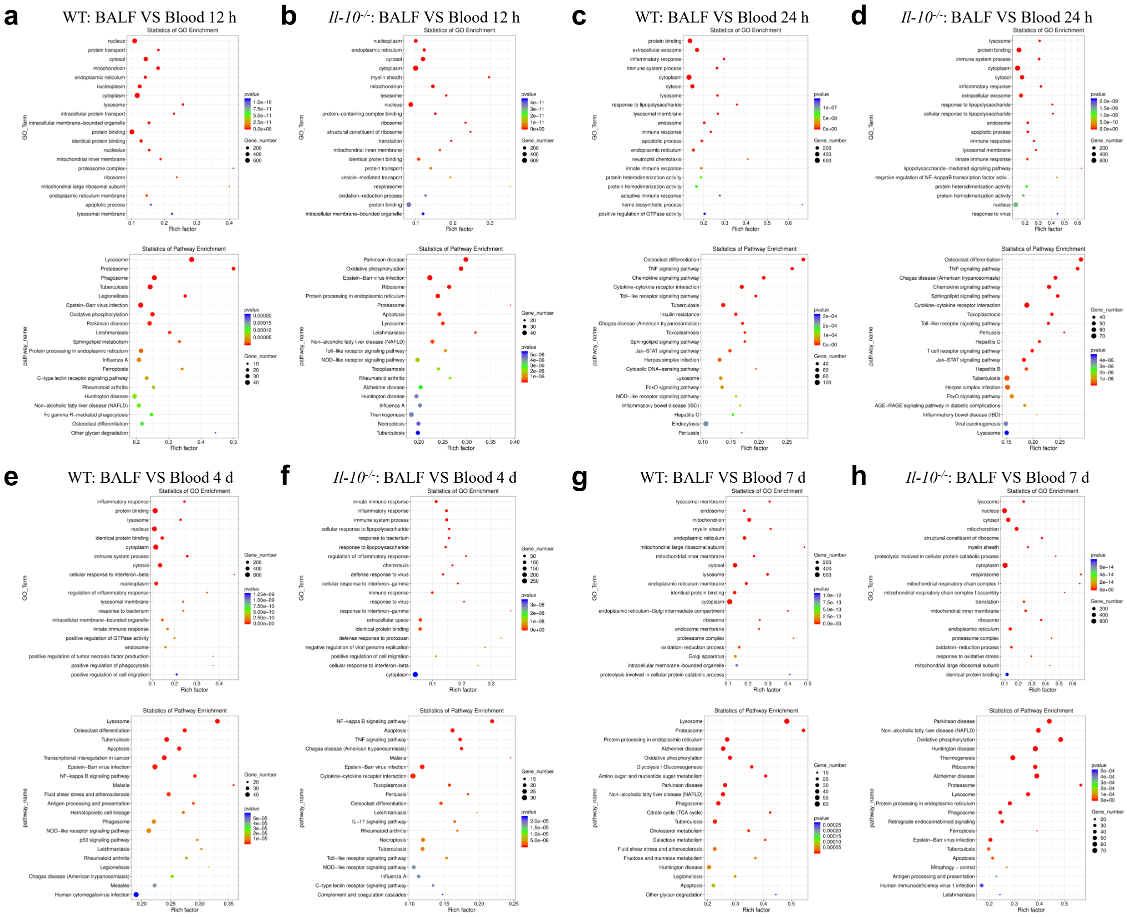

Supplementary Figure 14. **a-h** GO terms and KEGG pathway analyses of BALF and blood neutrophils in different treatment groups at different time points. *n* = 4 samples per group. VS, versus. The statistical analysis was performed by Fisher's test.

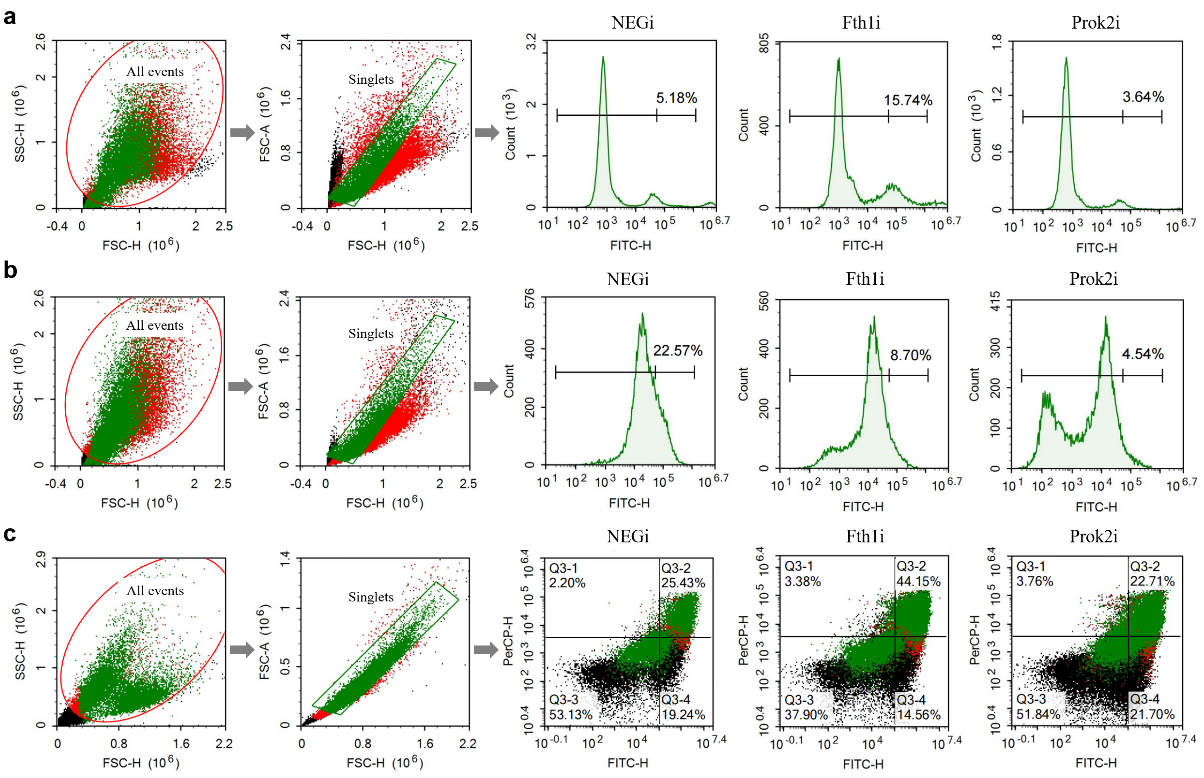

Supplementary Figure 15. **a-c** Gating strategy for the detection of ROS (**a**), phagocytosis (**b**) and apoptosis (**c**) of HL-60 cell-derived neutrophils.

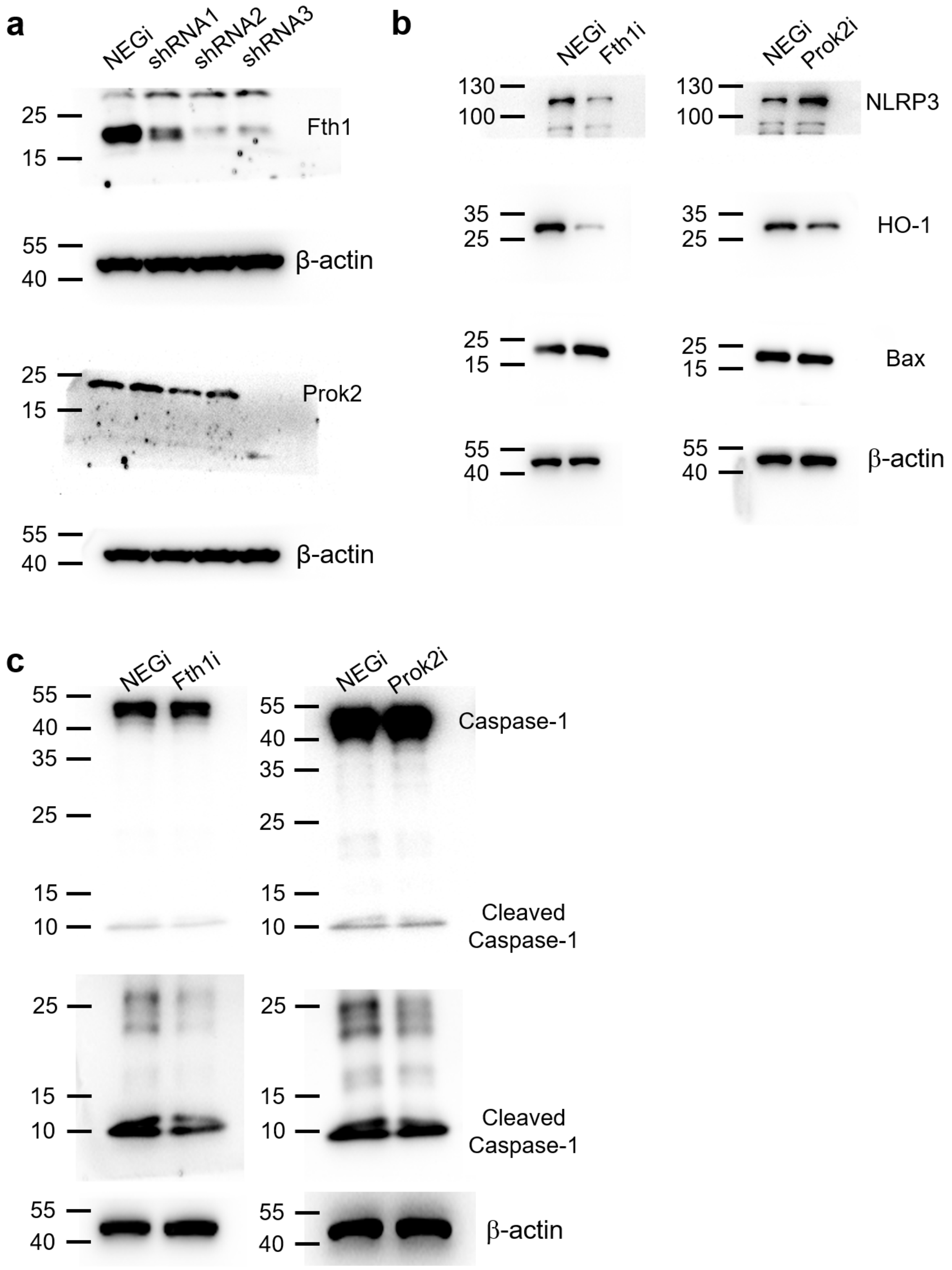

Supplementary Figure 16. **a-c** Uncropped scans of western blot with molecular weight markers, which are related with Supplementary Figure 10.

Supplementary Table 1: Demographic and clinical characteristics of 24 patients.

	Age, Yrs, (M, IQR)	Sex	Smoking	Pack-years, Yrs	Cancer history	Current disease	Sample	Underlying diseases					Outcome
								Hypertension	Diabetes	Cardiac disease	Autoimmune diseases	Others	Survival
**All% or median**	67(49-88)	62.50% Male	8.33%	-	16.67%	-	BALF	41.67%	12.5%	16.67%	4.17%	50.00%	75.00%
**Case 1**	49	F [a]	×	-	×	SP [c] Pneumonia		√	×	√	×	×	√
**Case 2**	54	M [b]	√	30	×			×	√	×	×	×	√
**Case 3**	66	F	×	-	√			×	×	×	×	×	√
**Case 4**	85	M	×	-	×	KP [d] Pneumonia		×	×	×	×	√	√
**Case 5**	64	F	×	-	×			×	√	×	×	×	√
**Case 6**	64	F	×	-	√			√	×	×	×	×	√
**Case 7**	71	M	×	-	√			×	×	×	×	×	√
**Case 8**	73	M	×	-	×	HI [e]		√	×	×	×	×	√
**Case 9**	70	M	×	-	×			×	×	×	×	√	√
**Case 10**	66	M	×	-	×			√	×	√	×	×	√
**Case 11**	64	F	×	-	√	Lung Cancer		×	×	×	×	×	√
**Case 12**	54	M	×	-	×			×	×	×	×	√	√
**Case 13**	87	F	×	-	×			×	×	×	×	×	√
**Case 14**	61	F	×	-	×	Pulmonary Fibrosis		×	×	×	×	√	√
**Case 15**	72	M	×	-	×			√	×	×	×	×	√
**Case 16**	67	F	×	-	×	AECOPD [f]		×	×	×	×	√	√
**Case 17**	54	M	√	30	×			×	×	×	×	√	√
**Case 18**	72	M	×	-	×	ARDS [g]		√	×	√	×	×	×
**Case 19**	71	M	×	-	×			×	√	×	√	√	×
**Case 20**	70	F	×	-	×			√	×	×	×	√	×
**Case 21**	65	M	×	-	×			×	×	×	×	√	×
**Case 22**	66	M	×	-	×			√	×	×	×	√	√
**Case 23**	88	M	×	-	×			√	×	×	×	√	×
**Case 24**	84	M	×	-	×			√	×	√	×	√	×

[a] F: Female. [b] M: Male. [c] SP: *Streptococcus pneumoniae*. [d] KP: *Klebsiella pneumoniae*. [e] HI: *Haemophilus influenzae*. [f] AECOPD: Acute exacerbation of chronic obstructive pulmonary disease. [g] ARDS: Acute respiratory distress syndrome.
