# Supplementary material for: Locally organised and activated Fth1hi neutrophils aggravate inflammation of acute lung injury in an IL-10-dependent manner
Source: Nat Commun. 2022 Dec 13;13:7703. doi: 10.1038/s41467-022-35492-y (PMC9745290; doi:10.1038/s41467-022-35492-y)
Supplement: Supplementary file 3 — Reporting Summary [file 41467_2022_35492_MOESM3_ESM.pdf]

nature portfolio

Corresponding author(s): Prof. Wei Gao

Last updated by author(s): Nov 11, 2022

Reporting Summary

Nature Portfolio wishes to improve the reproducibility of the work that we publish. This form provides structure for consistency and transparency in reporting. For further information on Nature Portfolio policies, see our Editorial Policies and the Editorial Policy Checklist.

Statistics

For all statistical analyses, confirm that the following items are present in the figure legend, table legend, main text, or Methods section.

n/a	Confirmed	
☐	☒	The exact sample size (n) for each experimental group/condition, given as a discrete number and unit of measurement
☐	☒	A statement on whether measurements were taken from distinct samples or whether the same sample was measured repeatedly
☐	☒	The statistical test(s) used AND whether they are one- or two-sided<br>*Only common tests should be described solely by name; describe more complex techniques in the Methods section.*
☒	☐	A description of all covariates tested
☐	☒	A description of any assumptions or corrections, such as tests of normality and adjustment for multiple comparisons
☐	☒	A full description of the statistical parameters including central tendency (e.g. means) or other basic estimates (e.g. regression coefficient) AND variation (e.g. standard deviation) or associated estimates of uncertainty (e.g. confidence intervals)
☐	☒	For null hypothesis testing, the test statistic (e.g. F, t, r) with confidence intervals, effect sizes, degrees of freedom and P value noted<br>*Give P values as exact values whenever suitable.*
☒	☐	For Bayesian analysis, information on the choice of priors and Markov chain Monte Carlo settings
☒	☐	For hierarchical and complex designs, identification of the appropriate level for tests and full reporting of outcomes
☐	☒	Estimates of effect sizes (e.g. Cohen's d, Pearson's r), indicating how they were calculated

Our web collection on statistics for biologists contains articles on many of the points above.

Software and code

Policy information about availability of computer code

Data collection	Single cell RNA sequencing of mouse lung samples was performed by 10× Genomics platform (USA). SMART-sequencing was conducted on the Illumina NovaseqTM 6000 (USA) platform using a 2×150 bp paired-end sequencing protocol.
Data analysis	Single cell RNA sequencing data were aligned to the mouse genome mm10 with Cell Ranger version 6.0 (10× Genomics, USA). The data were processed using the Seurat R package version 4.0 and Loupe Browser version 5.0.0. Images were processed with the ImageJ software (version 1.44p, National Institutes of Health, USA). Statistical analysis was performed by GraphPad Prism software (version 8.0.1, GraphPad Software, Inc., San Diego, CA).

For manuscripts utilizing custom algorithms or software that are central to the research but not yet described in published literature, software must be made available to editors and reviewers. We strongly encourage code deposition in a community repository (e.g. GitHub). See the Nature Portfolio guidelines for submitting code & software for further information.

Data

Policy information about availability of data

All manuscripts must include a data availability statement. This statement should provide the following information, where applicable:

- Accession codes, unique identifiers, or web links for publicly available datasets
- A description of any restrictions on data availability
- For clinical datasets or third party data, please ensure that the statement adheres to our policy

The raw data of single-cell RNA-seq generated in this study have been deposited in Genome Sequence Archive with accession ID CRA008837. The SMART-seq data are available under the accession ID CRA008856. The remaining data are available within the Article, Supplementary Information or Source Data files. Source data are provided with this paper.

Field-specific reporting

Please select the one below that is the best fit for your research. If you are not sure, read the appropriate sections before making your selection.

☒ Life sciences ☐ Behavioural & social sciences ☐ Ecological, evolutionary & environmental sciences

For a reference copy of the document with all sections, see nature.com/documents/nr-reporting-summary-flat.pdf

Life sciences study design

All studies must disclose on these points even when the disclosure is negative.

Sample size	In our study, a total of 18,146 cells from 5 mice (WT-PBS, n = 1 sample; WT-LPS, n = 2 samples; KO-LPS, n = 2 samples) were analyzed for transcriptional profiling, for which an average of 1,268 genes per cell were measured. SMART-sequencing was performed on 32 mice (n = 4 in each group). Sample size estimates has been conducted based on previous experience to obtain statistical significance and reproducibility. For in vivo studies, the sample size was determined to be sufficient to obtain the statistical difference between groups. For in vitro experiments, more than 3 samples were applied per group for minimal statistics requirements. For human studies, a total of 24 participants were enrolled for acquiring BALF samples. All experiments, except for the scRNA-seq and SMART-sequencing, were performed independently at least 3 times.
Data exclusions	For sc-RNA sequencing, cells with less than 500 detected genes, more than 25% percent mitochondria genes or UMI<500 were excluded.
Replication	All samples were biologically independent and three or more independent experiments with similar results were performed, except for the sequencing data. Because the abundance of airway and blood neutrophil subsets from mice were relatively low.
Randomization	All mice and tissue samples grouped randomly and blindly, except the genotype factors. Because we determined the genotype before the experiments.
Blinding	In this research, all analysis were performed blind. The investigators were blinded to group allocation during data collection. Data analysis were performed by different researchers responsible for genotyping and analysis to avoid conscious and unconscious bias.

Behavioural & social sciences study design

All studies must disclose on these points even when the disclosure is negative.

Study description	*Briefly describe the study type including whether data are quantitative, qualitative, or mixed-methods (e.g. qualitative cross-sectional, quantitative experimental, mixed-methods case study).*
Research sample	*State the research sample (e.g. Harvard university undergraduates, villagers in rural India) and provide relevant demographic information (e.g. age, sex) and indicate whether the sample is representative. Provide a rationale for the study sample chosen. For studies involving existing datasets, please describe the dataset and source.*
Sampling strategy	*Describe the sampling procedure (e.g. random, snowball, stratified, convenience). Describe the statistical methods that were used to predetermine sample size OR if no sample-size calculation was performed, describe how sample sizes were chosen and provide a rationale for why these sample sizes are sufficient. For qualitative data, please indicate whether data saturation was considered, and what criteria were used to decide that no further sampling was needed.*
Data collection	*Provide details about the data collection procedure, including the instruments or devices used to record the data (e.g. pen and paper, computer, eye tracker, video or audio equipment) whether anyone was present besides the participant(s) and the researcher, and whether the researcher was blind to experimental condition and/or the study hypothesis during data collection.*
Timing	*Indicate the start and stop dates of data collection. If there is a gap between collection periods, state the dates for each sample cohort.*
Data exclusions	*If no data were excluded from the analyses, state so OR if data were excluded, provide the exact number of exclusions and the rationale behind them, indicating whether exclusion criteria were pre-established.*
Non-participation	*State how many participants dropped out/declined participation and the reason(s) given OR provide response rate OR state that no participants dropped out/declined participation.*
Randomization	*If participants were not allocated into experimental groups, state so OR describe how participants were allocated to groups, and if allocation was not random, describe how covariates were controlled.*

Ecological, evolutionary & environmental sciences study design

All studies must disclose on these points even when the disclosure is negative.

Study description	*Briefly describe the study. For quantitative data include treatment factors and interactions, design structure (e.g. factorial, nested, hierarchical), nature and number of experimental units and replicates.*
Research sample	*Describe the research sample (e.g. a group of tagged Passer domesticus, all Stenocereus thurberi within Organ Pipe Cactus National Monument), and provide a rationale for the sample choice. When relevant, describe the organism taxa, source, sex, age range and any manipulations. State what population the sample is meant to represent when applicable. For studies involving existing datasets, describe the data and its source.*
Sampling strategy	*Note the sampling procedure. Describe the statistical methods that were used to predetermine sample size OR if no sample-size calculation was performed, describe how sample sizes were chosen and provide a rationale for why these sample sizes are sufficient.*
Data collection	*Describe the data collection procedure, including who recorded the data and how.*
Timing and spatial scale	*Indicate the start and stop dates of data collection, noting the frequency and periodicity of sampling and providing a rationale for these choices. If there is a gap between collection periods, state the dates for each sample cohort. Specify the spatial scale from which the data are taken*
Data exclusions	*If no data were excluded from the analyses, state so OR if data were excluded, describe the exclusions and the rationale behind them, indicating whether exclusion criteria were pre-established.*
Reproducibility	*Describe the measures taken to verify the reproducibility of experimental findings. For each experiment, note whether any attempts to repeat the experiment failed OR state that all attempts to repeat the experiment were successful.*
Randomization	*Describe how samples/organisms/participants were allocated into groups. If allocation was not random, describe how covariates were controlled. If this is not relevant to your study, explain why.*
Blinding	*Describe the extent of blinding used during data acquisition and analysis. If blinding was not possible, describe why OR explain why blinding was not relevant to your study.*

Did the study involve field work? ☐ Yes ☐ No

Field work, collection and transport

Field conditions	*Describe the study conditions for field work, providing relevant parameters (e.g. temperature, rainfall).*
Location	*State the location of the sampling or experiment, providing relevant parameters (e.g. latitude and longitude, elevation, water depth).*
Access & import/export	*Describe the efforts you have made to access habitats and to collect and import/export your samples in a responsible manner and in compliance with local, national and international laws, noting any permits that were obtained (give the name of the issuing authority, the date of issue, and any identifying information).*
Disturbance	*Describe any disturbance caused by the study and how it was minimized.*

Reporting for specific materials, systems and methods

We require information from authors about some types of materials, experimental systems and methods used in many studies. Here, indicate whether each material, system or method listed is relevant to your study. If you are not sure if a list item applies to your research, read the appropriate section before selecting a response.

Materials & experimental systems

n/a	Involved in the study	
☐	☒	Antibodies
☐	☒	Eukaryotic cell lines
☒	☐	Palaeontology and archaeology
☐	☒	Animals and other organisms
☐	☒	Human research participants
☒	☐	Clinical data
☒	☐	Dual use research of concern

Methods

n/a	Involved in the study	
☒	☐	ChIP-seq
☐	☒	Flow cytometry
☒	☐	MRI-based neuroimaging

Antibodies

Antibodies used

1. Anti-Ferritin Heavy Chain antibody (Rabbit polyclonal to Ferritin Heavy Chain), Abcam, cat.#ab65080, IHC: 1:200, IF: 1:200, WB: 1:1000.
2. Anti-Ly6g + Ly6c antibody (Rat monoclonal [RB6-8C5] to Ly6g + Ly6c), Abcam, cat.#ab25377, IHC: 1:100, IF: 1:100.
3. Anti-Neutrophil Elastase antibody (Rabbit polyclonal to Neutrophil Elastase), Abcam, cat.#ab68672, IHC: 1:100.
4. Anti-Myeloperoxidase antibody (Rabbit monoclonal [EPR20257] to Myeloperoxidase), Abcam, cat.#ab20867, IHC: 1:1000.
5. Anti-Prokineticin 2/PK2 antibody (Rabbit polyclonal to Prokineticin 2), Zen Bio, cat.#506705, IF: 1:500, WB: 1:1000.
6. Anti-Prokineticin 2/PK2 antibody (Rabbit polyclonal to Prokineticin 2/PK2), Abcam, cat.#ab76747, IF: 1:100, WB: 1:1000.
7. FITC Rat anti-Mouse Ly6G Flow cytometry antibody, BD Pharmingen, cat.#: 551460. Flow cytometry: 1:200.
8. Secondary goat anti-rabbit IgG antibody conjugated with Cy3, Servicebio, cat.#GB21303, IF: 1:300.
9. Secondary goat anti-rat IgG antibody conjugated with FITC, Servicebio, cat.#GB22302, IF: 1:100.
10. Anti-NLRP3 (D4D8T) Rabbit mAb, Cell Signaling Technology, cat.#15101, WB: 1:1000.
11. Anti-pro Caspase-1 + p10 + p12 antibody (Rabbit monoclonal [EPR16883] to pro Caspase-1 + p10 + p12), Abcam, cat.#ab179515, WB: 1:1000.
12. Anti-HO-1 (D60G11) Rabbit mAb, Cell Signaling Technology, cat.#5853, WB: 1:1000.
13. Anti-Bax Rabbit pAb, Zen Bio, cat.#380709, WB: 1:1000.
14. Anti-β-Actin (8H10D10) Mouse mAb, Cell Signaling Technology, cat.#3700, WB: 1:1000.
15. Anti-mouse IgG, HRP-linked Antibody, Cell Signaling Technology, cat.#7076, WB:1:1000.
16. Anti-rabbit IgG, HRP-linked Antibody, Cell Signaling Technology, cat.#7074, WB:1:1000.

Validation

1. https://www.abcam.cn/ferritin-heavy-chain-antibody-ab65080.html.
2. https://www.abcam.cn/ly6g--ly6c-antibody-rb6-8c5-ab25377.html.
3. https://www.abcam.cn/neutrophil-elastase-antibody-ab68672.html.
4. https://www.abcam.cn/myeloperoxidase-antibody-epr20257-ab208670.html.
5. The antibody has now been discontinued.
6. https://www.abcam.cn/prokineticin-2pk2-antibody-ab76747.html.
7. https://www.bdbiosciences.com/zh-cn/products/reagents/flow-cytometry-reagents/research-reagents/single-color-antibodies-ruo/fitc-rat-anti-mouse-ly-6g.551460.
8. https://www.servicebio.cn/goodsdetail?id=253.
9. https://www.servicebio.cn/goodsdetail?id=258.
10. https://www.cellsignal.cn/products/primary-antibodies/nlrp3-d4d8t-rabbit-mab/15101?site-search-type=Products&N=4294956287&Ntt=15101&fromPage=plp&_requestid=476622.
11. https://www.abcam.cn/pro-caspase-1--p10--p12-antibody-epr16883-ab179515.html.
12. https://www.cellsignal.cn/products/primary-antibodies/ho-1-d60g11-rabbit-mab/5853?site-search-type=Products&N=4294956287&Ntt=5853&fromPage=plp&_requestid=479533.
13. http://www.zen-bio.cn/prod_view.aspx?IsActiveTarget=True&TypeId=180&Id=543189&FId=t3:180:3.
14. https://www.cellsignal.cn/products/primary-antibodies/b-actin-8h10d10-mouse-mab/3700?site-search-type=Products&N=4294956287&Ntt=3700&fromPage=plp&_requestid=480717.
15. https://www.cellsignal.cn/products/secondary-antibodies/anti-mouse-igg-hrp-linked-antibody/7076?Ns=productCitationsCount%7C1&N=0+4294960093+102287+4294956287&fromPage=plp.
16. https://www.cellsignal.cn/products/secondary-antibodies/anti-rabbit-igg-hrp-linked-antibody/7074?Ns=productCitationsCount%7C1&N=0+4294960093+102287+4294956287&fromPage=plp.

The validation of all of the antibodies depends on product datasheet and published literature.

Eukaryotic cell lines

Policy information about cell lines

Cell line source(s)	Human promyelocytic leukemia (HL-60) cells.
Authentication	Identity of the cell line was frequently checked by its morphological features.
Mycoplasma contamination	The cell line was mycoplasma-negative.
Commonly misidentified lines (See ICLAC register)	No commonly misidentified cell lines are used in this study.

Palaeontology and Archaeology

Specimen provenance	*Provide provenance information for specimens and describe permits that were obtained for the work (including the name of the issuing authority, the date of issue, and any identifying information). Permits should encompass collection and, where applicable, export.*
Specimen deposition	*Indicate where the specimens have been deposited to permit free access by other researchers.*
Dating methods	*If new dates are provided, describe how they were obtained (e.g. collection, storage, sample pretreatment and measurement), where they were obtained (i.e. lab name), the calibration program and the protocol for quality assurance OR state that no new dates are provided.*

☐ Tick this box to confirm that the raw and calibrated dates are available in the paper or in Supplementary Information.

Ethics oversight	*Identify the organization(s) that approved or provided guidance on the study protocol, OR state that no ethical approval or guidance was required and explain why not.*

Note that full information on the approval of the study protocol must also be provided in the manuscript.

Animals and other organisms

Policy information about studies involving animals; ARRIVE guidelines recommended for reporting animal research

Laboratory animals	B6.129P2-Il10tm1/Nju (IL-10-/-) and control (C57BL/6JNju; WT) mice were used in most of the in vivo studies: both male and female ranging from 6-8 weeks of age. In some experiments, male C57BL/6 mice (6-8 weeks old) were used. Mice were housed under specific pathogen-free conditions and in a standarded light-dark cycle with food and water ad libitum.
Wild animals	The study did not involve wild animals.
Field-collected samples	The study did not involve samples collected from the field.
Ethics oversight	All animal experiments were performed according to the guidelines of Shanghai Committee for Accreditation of Laboratory Animal, and the research protocol was approved by the Laboratory Animal Research Center Review Board of Tongji University (Permit Number: TJBB03721106) (Shanghai, China). All surgery was performed under pentobarbital sodium anesthesia with efforts to minimize animal suffering.

Note that full information on the approval of the study protocol must also be provided in the manuscript.

Human research participants

Policy information about studies involving human research participants

Population characteristics	A total of 24 patients (15 males and 9 females; range, 49-88 years old) were enrolled in our research, among which 7 developed into ARDS within 48h after admission. The demographic and clinical characteristics were provided in Supplemental Table 1.
Recruitment	All patients at risk for the development of ARDS could be asked to be recruited. All patients provided written consent to participate in the study approval of local medical ethnics.
Ethics oversight	The study was approved by the ethics commission of Shanghai East Hospital, China (Permit Number: 2019YYS138), and informed consent was obtained from all participants.

Note that full information on the approval of the study protocol must also be provided in the manuscript.

Clinical data

Policy information about clinical studies

All manuscripts should comply with the ICMJE guidelines for publication of clinical research and a completed CONSORT checklist must be included with all submissions.

Clinical trial registration	*Provide the trial registration number from ClinicalTrials.gov or an equivalent agency.*
Study protocol	*Note where the full trial protocol can be accessed OR if not available, explain why.*
Data collection	*Describe the settings and locales of data collection, noting the time periods of recruitment and data collection.*
Outcomes	*Describe how you pre-defined primary and secondary outcome measures and how you assessed these measures.*

Dual use research of concern

Policy information about dual use research of concern

Hazards

Could the accidental, deliberate or reckless misuse of agents or technologies generated in the work, or the application of information presented in the manuscript, pose a threat to:

No	Yes	
☐	☐	Public health
☐	☐	National security
☐	☐	Crops and/or livestock
☐	☐	Ecosystems
☐	☐	Any other significant area

Experiments of concern

Does the work involve any of these experiments of concern:

No	Yes	
☐	☐	Demonstrate how to render a vaccine ineffective
☐	☐	Confer resistance to therapeutically useful antibiotics or antiviral agents
☐	☐	Enhance the virulence of a pathogen or render a nonpathogen virulent
☐	☐	Increase transmissibility of a pathogen
☐	☐	Alter the host range of a pathogen
☐	☐	Enable evasion of diagnostic/detection modalities
☐	☐	Enable the weaponization of a biological agent or toxin
☐	☐	Any other potentially harmful combination of experiments and agents

ChIP-seq

Data deposition

☐ Confirm that both raw and final processed data have been deposited in a public database such as GEO.

☐ Confirm that you have deposited or provided access to graph files (e.g. BED files) for the called peaks.

Data access links<br>*May remain private before publication.*	*For "Initial submission" or "Revised version" documents, provide reviewer access links. For your "Final submission" document, provide a link to the deposited data.*
Files in database submission	*Provide a list of all files available in the database submission.*
Genome browser session<br>(e.g. UCSC)	*Provide a link to an anonymized genome browser session for "Initial submission" and "Revised version" documents only, to enable peer review. Write "no longer applicable" for "Final submission" documents.*

Methodology

Replicates	*Describe the experimental replicates, specifying number, type and replicate agreement.*
Sequencing depth	*Describe the sequencing depth for each experiment, providing the total number of reads, uniquely mapped reads, length of reads and whether they were paired- or single-end.*
Antibodies	*Describe the antibodies used for the ChIP-seq experiments; as applicable, provide supplier name, catalog number, clone name, and lot number.*
Peak calling parameters	*Specify the command line program and parameters used for read mapping and peak calling, including the ChIP, control and index files used.*
Data quality	*Describe the methods used to ensure data quality in full detail, including how many peaks are at FDR 5% and above 5-fold enrichment.*
Software	*Describe the software used to collect and analyze the ChIP-seq data. For custom code that has been deposited into a community repository, provide accession details.*

Flow Cytometry

Plots

Confirm that:

☒ The axis labels state the marker and fluorochrome used (e.g. CD4-FITC).

☒ The axis scales are clearly visible. Include numbers along axes only for bottom left plot of group (a 'group' is an analysis of identical markers).

☒ All plots are contour plots with outliers or pseudocolor plots.

☒ A numerical value for number of cells or percentage (with statistics) is provided.

Methodology

Sample preparation	After anesthetization, mice underwent disinfection and tracheotomy. Ice-cold PBS (0.8 mL) was slowly injected through the trachea, followed by carefully withdraw. The volume of the retrieved fluid (BALF) was>70% of the injected volume. BALF samples were centrifuged at 1200 rpm for 10 min at 4°C to collect BAL inflammatory cells. Polychromatic flow cytometry was also performed to identify the apoptosis rate of neutrophils in the BALF. The cells were washed in PBS and stained with a cocktail of antibodies against phycoerythrin (PE)-Annexin V and fluorescein isothiocyanate (FITC)-Ly6G. After incubation for

15 min at room temperature in the dark, the cells were washed and stained with 7-Amino-Actinomycin (7-AAD).
For HL-60 cell line, cells were incubated with 10 μM 2',7'-dichlorofluorescein diacetate (DCFH-DA) fluorescent probe in the dark for 30 min, rinsed thoroughly and collected for flow cytometry. As for phagocytosis assay, cells were incubated with phenol red-free medium containing 1 mg/mL pHrodo Green E. coli BioParticles conjugate for 1 h at 37°C , 5% CO_2.
Afterwards, samples were collected for flow cytometry and the fluorescence intensity was analyzed for phagocytosis.

Instrument

Flow cytometer (BD Accuri C6, USA), flow cytometer (Attune NxT, Invitrogen, USA).

Software

BD Accuri C6 Software, Attune NxT Software.

Cell population abundance

For in vivo sorted airway neutrophils from ALI mice, BALF cells are abundance of Ly6G, which is high > 80% of total cells.

Gating strategy

From FSC-A/SSC-A, cell debris were eliminated, and from FSC-A/FSC-H single cells were selected. Cells were then gated on FITC: Ly6G+ cells (neutrophils). The apoptosis ratio of FITC-Ly6G+ neutrophils was calculated as PE-Annexin V+/7-AAD+ and PE-Annexin V+/7-AAD-.
For HL-60 cell line, cells were gated on the FSC/SSC plots, then single cells were gated on the FSC-H/FSC-A plots. Interested cells were stained with DCFH-DA fluorescent probe or pHrodo Green E. coli BioParticles conjugate. Gating was determined by blank and single color-staining. Besides, the apoptosis ratio of HL-60 cells was also calculated as PE-Annexin V+/7-AAD+ and PE-Annexin V+/7-AAD-.

☒ Tick this box to confirm that a figure exemplifying the gating strategy is provided in the Supplementary Information.

Magnetic resonance imaging

Experimental design

Design type

Indicate task or resting state; event-related or block design.

Design specifications

Specify the number of blocks, trials or experimental units per session and/or subject, and specify the length of each trial or block (if trials are blocked) and interval between trials.

Behavioral performance measures

State number and/or type of variables recorded (e.g. correct button press, response time) and what statistics were used to establish that the subjects were performing the task as expected (e.g. mean, range, and/or standard deviation across subjects).

Acquisition

Imaging type(s)

Specify: functional, structural, diffusion, perfusion.

Field strength

Specify in Tesla

Sequence & imaging parameters

Specify the pulse sequence type (gradient echo, spin echo, etc.), imaging type (EPI, spiral, etc.), field of view, matrix size, slice thickness, orientation and TE/TR/flip angle.

Area of acquisition

State whether a whole brain scan was used OR define the area of acquisition, describing how the region was determined.

Diffusion MRI ☐ Used ☐ Not used

Preprocessing

Preprocessing software

Provide detail on software version and revision number and on specific parameters (model/functions, brain extraction, segmentation, smoothing kernel size, etc.).

Normalization

If data were normalized/standardized, describe the approach(es): specify linear or non-linear and define image types used for transformation OR indicate that data were not normalized and explain rationale for lack of normalization.

Normalization template

Describe the template used for normalization/transformation, specifying subject space or group standardized space (e.g. original Talairach, MNI305, ICBM152) OR indicate that the data were not normalized.

Noise and artifact removal

Describe your procedure(s) for artifact and structured noise removal, specifying motion parameters, tissue signals and physiological signals (heart rate, respiration).

Volume censoring

Define your software and/or method and criteria for volume censoring, and state the extent of such censoring.

Statistical modeling & inference

Model type and settings

Specify type (mass univariate, multivariate, RSA, predictive, etc.) and describe essential details of the model at the first and second levels (e.g. fixed, random or mixed effects; drift or auto-correlation).

Effect(s) tested

Define precise effect in terms of the task or stimulus conditions instead of psychological concepts and indicate whether ANOVA or factorial designs were used.

Specify type of analysis: ☐ Whole brain ☐ ROI-based ☐ Both

Statistic type for inference (See Eklund et al. 2016)	*Specify voxel-wise or cluster-wise and report all relevant parameters for cluster-wise methods.*
Correction	*Describe the type of correction and how it is obtained for multiple comparisons (e.g. FWE, FDR, permutation or Monte Carlo).*

Models & analysis

n/a	Involved in the study
☐	☐ Functional and/or effective connectivity
☐	☐ Graph analysis
☐	☐ Multivariate modeling or predictive analysis

Functional and/or effective connectivity	*Report the measures of dependence used and the model details (e.g. Pearson correlation, partial correlation, mutual information).*
Graph analysis	*Report the dependent variable and connectivity measure, specifying weighted graph or binarized graph, subject- or group-level, and the global and/or node summaries used (e.g. clustering coefficient, efficiency, etc.).*
Multivariate modeling and predictive analysis	*Specify independent variables, features extraction and dimension reduction, model, training and evaluation metrics.*
